# Accurate detection of intracranial extension of jugulotympanic paraganglioma by [^18^F]FDOPA-PET/CT comparing to MRI

**DOI:** 10.1007/s00259-021-05490-1

**Published:** 2021-08-11

**Authors:** Zahra Jamshidi Araghi, Shaghayegh Ranjbar, Michael Paar, Christian Pirich, Mohsen Beheshti

**Affiliations:** 1 Division of Molecular Imaging and Theranostics, Department of Nuclear Medicine, University Hospital Salzburg, Paracelsus Medical University, Muellner Hauptstrasse 48, 5020 Salzburg, Austria; 2 Department of Radiology, University Hospital Salzburg, Paracelsus Medical University, Salzburg, Austria

Jugulotympanic paraganglioma (JTP) is a rare, slow-growing, and usually benign tumor in the jugular foramen [1, 2]. Magnetic resonance imaging (MRI) is used as modality of choice for assessment of JTP [3, 4]. Positron emission tomography–computed tomography (PET/CT) using specific radiotracers (e.g., ^68^ Ga-DOTA-derivatives) have been performed for localization of multifocal JTP or sites of metastases in malignant tumors [5, 6]. However, the impact of PET/CT using [18F]fluorodihydroxyphenylalanin ([18F]FDOPA), as a specific radiotracer for neuroendocrine tumors (NET), has been less presented in the literature. This particular case presents the superior value of [^18^F]FDOPA-PET/CT comparing to MRI for depiction of the entire tumor extent in a 53-year-old woman presented with progressive symptoms of vertigo and recurrent tinnitus on the left side for 6 months. Brain MRI reported an osteodestructive mass in the area of the left jugular foramen, extending caudally along the cervical nerve sheath up to the level of hyoid (e–h, arrows).

[^18^F]FDOPA-PET/CT has more accurately defined the intracranial extension of the JPT showing a diffuse, intense tracer uptake in the left jugular foramen, extending to the ipsilateral sigmoid sinus, with tumor growth along the left cerebellar hemisphere up to the lateral aspect of cerebellar tentorium and caudally along the cervical vascular nerve sheath to the level of the hyoid bone (SUV max. 26.3) (a–d. arrows). JPT was approved by histopathology.

A physiologic intense [^18^F]FDOPA-PET/CT uptake was also seen in the gallbladder as excretory organ (a and b, green arrow).

The present case emphasizes again on the added value of functional PET imaging using specific radiotracers for the accurate assessment of JPTs, especially when performing in a hybrid PET/MRI setting.

**Figure:**
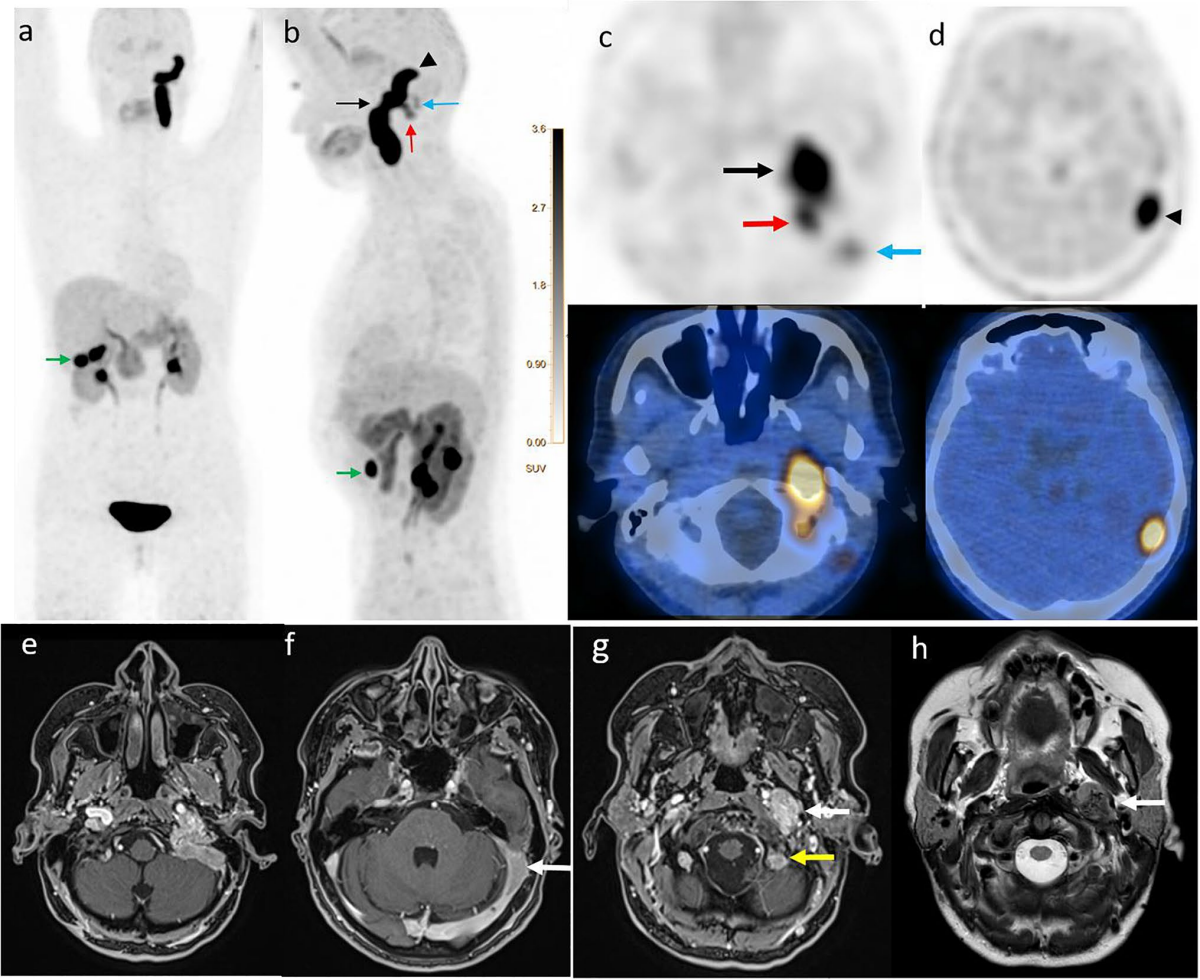
**a**. [18F]FDOPA PET: MIP (Maximum Intensity Projection) anterior view shows intensive tracer uptake along the left cervical and intracranial regions. **b**. [18F]FDOPA PET/: MIP left lateral **c–d**. [18F]FDOPA PET (upper row) and fused PET/CT axial images (lower row) show intensive tracer uptake in the area of the left jugular foramen (**b–c**, black arrow) with cranial extension to the ipsilateral sigmoid sinus, and condylar emissary vein (**b–c**, red arrow), and an intracranial extension along the left cerebellar hemisphere to the lateral aspect of cerebellar tentorium (**b–d**, arrowhead). In the caudal dimension, the tumoral tissue shows intensive tracer uptake along the cervical vascular nerve sheath to the level of the hyoid bone. In addition, a small tumoral extension with faint uptake is also seen in the splenius capitis muscle region (**b–c**, blue arrow). **e–h**. Magnetic Resonance Imaging (MRI) axial T1 fat suppression with intravenous Gadolinium (**e–g**), axial T2 TSE (**h**) demonstrating large tumour expansion centred at the jugular foramen with local bony destruction. Contrast enhancement with typical salt and pepper-appearance with multiple internal flow voids are best seen in T2. Tumoral extension is evident in the sigmoid sinus (**f**, arrow), the condylar emissary vein via the condylar canal (**g**, yellow arrow) as well extra cranially along the internal jugular vein with encasement of the internal carotid artery (**g–h**, white arrow)
